# Four-Wire Interface ASIC for a Multi-Implant Link

**DOI:** 10.1109/TCSI.2017.2731659

**Published:** 2017-08-15

**Authors:** Sara S. Ghoreishizadeh, Dorian Haci, Yan Liu, Nick Donaldson, Timothy G. Constandinou

**Affiliations:** 1 Department of Electrical and Electronic EngineeringImperial College London London SW7 2AZ U.K.; 2 Department of Medical Physics and Biomedical EngineeringUniversity College London London WC1E 6BT U.K.

**Keywords:** Biotelemetry, implantable, link, wireline, full-duplex, communication

## Abstract

This paper describes an on-chip interface for recovering power and providing full-duplex communication over an AC-coupled 4-wire lead between active implantable devices. The target application requires two modules to be implanted in the brain (cortex) and upper chest; connected via a subcutaneous lead. The brain implant consists of multiple identical “optrodes” that facilitate a bidirectional neural interface (electrical recording and optical stimulation), and the chest implant contains the power source (battery) and processor module. The proposed interface is integrated within each optrode ASIC allowing full-duplex and fully-differential communication based on Manchester encoding. The system features a head-to-chest uplink data rate (up to 1.6 Mbps) that is higher than that of the chest-to-head downlink (100 kbps), which is superimposed on a power carrier. On-chip power management provides an unregulated 5-V dc supply with up to 2.5-mA output current for stimulation, and two regulated voltages (3.3 and 3 V) with 60-dB power supply rejection ratio for recording and logic circuits. The 4-wire ASIC has been implemented in a 0.35-$\mu \text{m}$ CMOS technology, occup-ying a 1.5-mm^2^ silicon area, and consumes a quiescent current of $91.2~\mu \text{A}$. The system allows power transmission with measured efficiency of up to 66% from the chest to the brain implant. The downlink and uplink communication are successfully tested in a system with two optrodes and through a 4-wire implantable lead.

## I. Introduction

Neural Prostheses have experienced significant progress in the last two decades [1], generating real impact particularly in clinical applications such as cochlear implants and deep brain stimulation (DBS) therapy. Basic research and new translational efforts are now developing Brain Machine Interfaces (BMIs) and closed loop therapies for epilepsy and Parkinson’s [2]–[4].

These advances are in part due to ever improving micro-technology, now allowing integrated *implantable medical devices* (IMDs) to be ultra compact and require ultra low power [5]. Even so, the energy source remains a key challenge; requiring either a large-capacity implanted battery, or an externally worn battery with wireless power transmission (or a combination of the two). An implanted battery is required if the medical device is serving a life critical function, for example, pacemaker or implantable defibrillator. Then, depending on the energy budget either a high capacity (non-rechargeable) primary cell or low capacity rechargeable battery is used. Rechargeable batteries typically have a lifetime of a few thousand charge cycles, but have significantly less energy capacity (per unit volume) than non-rechargeable cells [6].

A second key challenge is how to achieve reliable and efficient communication. Implanted devices require reliable means for wireless data transfer for calibration and real-time control purposes and/or communicating sensor/actuator/stimulation data. Clinical devices currently only have a single active implant. The communication required here thus tends to be by a transcutaneous inductive link [7] to an external programmer or charger. Next generation devices however, are developing multi-implant active modules [8] that will additionally require intra-body communication and also a means of sharing power [9].

Although intra-body communication can be achieved using wireless methods (e.g. electromagnetic, ultrasound, capacitive, etc) [10]–[12], a wired link has the additional advantage in that it can also be used to transmit/share a power source. Challenges however with an implanted lead include: mechanical stress, MRI safety/compatibility, and electrical properties (e.g. load impedance affecting power dissipation, communication bandwidth, etc). The presence of any static DC electric field(s) in or across an implanted cable is highly undesirable. This can accelerate corrosion and eventually cause failure [13]–[15], compromising patient safety if, for example, the lead is broken or a connector leaks current.

This work proposes a new protocol for a wired link that achieves both power transfer and data communication using four conductors. The circuit design of the 4-wire interface was first introduced in [16]. In this paper we describe the complete fabricated system and present silicon verified measurements of an end-to-end platform including an implantable lead. This paper is organized as follows: Section II introduces the multi-implant system concept and key requirements; Section III describes the communication channel (4-wire lead); Section IV analyses the power transfer efficiency; Section V details the circuit implementation; Section VI presents the fabricated system; Section VII presents silicon verified measurements and Section VIII concludes this work.

## II. System Concept

**Fig. 1.**
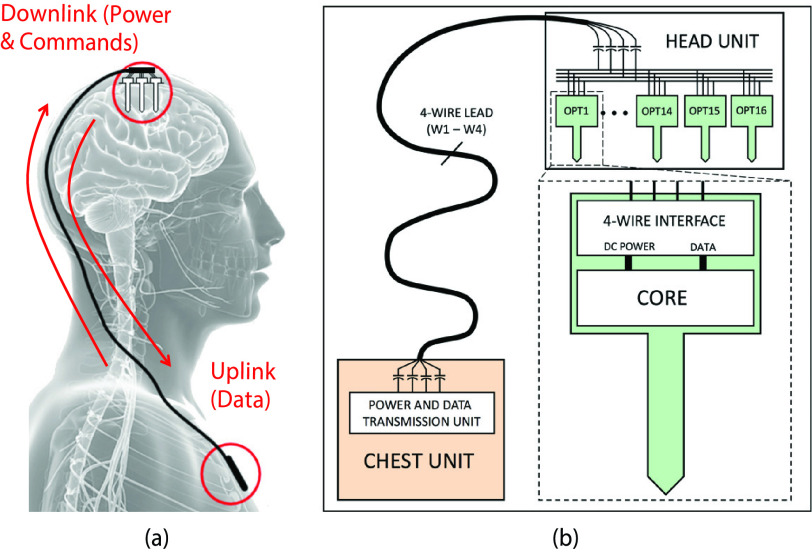
System concept showing: (a) subcutaneous lead between implanted chest and head units; (b) detail of 4-wire connectivity at each end of the lead.

The system concept of the multi-implant system is illustrated in Fig. 1. The chest implant includes the energy source (rechargeable battery) and processor unit, for powering and controlling the brain implant. The brain implant contains an optrode array (opto-electrodes) for observing neural activity (electrical recording), and neuromodulation (optical stimulation using optogenetics). The 4-wire interface facilitates the following functions: (i) providing power from the chest implant to the brain implant; (ii) communicating configuration data and stimulation control commands via a downlink; (iii) receiving recording and diagnostic data via an uplink. Full-duplex communication is required for simultaneous data recording and stimulation.

In the brain implant, a passive baseplate hosts multiple identical optrodes, a few discrete components and connects to the 4-wire lead. Each optrode has a number of electrical recording sites (via electrodes) to provide spatial resolution for Local Field Potential (LFP) recording, and a number of optical stimulation sites (via $\mu $LEDs) for optical stimulation [17]. The neural interface optrodes are fully-integrated in CMOS technology, described in more detail in [2] and [18].

In order to reduce the risk of corrosion to lead-insulation due to any static electric fields or possibility of any current leakage, all connections outside the chest unit and optrodes have DC-blocking capacitors placed in series with the wires. To keep the number of wires to a minimum, all wires are shared and connected in parallel to all optrode devices.

Although a wired link can provide improved efficiency and increased distance of transmission compared to wireless methods, the currently available leads (approved for human use) impose limitations on the system due to their relatively low bandwidth, large transmission delay, high wire-to-wire capacitances and non-uniform characteristics (e.g. each conductor has different parasitics) [19], [20]. These also tend to be unshielded to improve mechanical properties (e.g. flexibility). We have therefore adopted a fully-differential power and data transmission scheme for robustness to common-mode noise and lead interference. Furthermore, to minimize the number of wires and save energy (i.e. chest implant battery-life), the power transmission and downlink (i.e. chest-to-brain) are combined on a single pair of wires.

The uplink (brain-to-chest) can also be combined with the downlink/power wire pair to further reduce the number of wires – Load Shift Key can be performed by individual implants in a time multiplexing manner. However, the superimposed uplink data would be virtually undetectable at the chest unit because of in-band interference due to all the optrodes contributing noise. Moreover, the different optrodes would present a different load impedance; changing dynamically according to the stimulation mode and intensity. This would require complex signal processing at the chest unit to recover the uplink data. Therefore, and to ensure a reliable data recovery on the chest implant, we opted for uplink communication through a separate pair of wires within the same lead.

To avoid the need for a large off-chip smoothing capacitor at the AC-DC conversion for each optrode, a square-wave was chosen (instead of sinusoidal) for power transmission in the downlink. Both the down- and uplink communications use baseband digital modulation due to relatively small bandwidth of the lead wires. The downlink waveform amplitude is amplified by the chest unit to allow AC-power transmission to the optrodes. The optrodes access the channel for uplink communication in a Time Domain Multiple Access (TDMA) mode. To enable TDMA, the optrodes are synchronized with the chest implant. This is achieved on each optrode by recovering the clock in addition to commands from the downlink. In addition to the TDMA, other operation modes such as handshake are embedded, for example, for test diagnostics on each optrode and system start-up. Here the Finite State Machine (FSM) on the optrode may choose to use a lower clock frequency. In order to facilitate the uplink data recovery on the chest unit, clock is also transmitted with uplink data. Manchester coding was chosen for combining data and clock on both downlink and uplink.

Key design parameters here are the frequency and amplitude of the voltage waveforms on each wire. These are determined as follows: first, to ensure minimal energy loss in the wires, both frequency and amplitude are minimized. Then, since the power and downlink data are combined, the power transmission frequency is chosen based on the downlink datarate requirement. The downlink data rate is determined by the command packet size and frequency. The commands are to determine the stimulation frequency, location, and intensity as well as optrode configuration and diagnostics. The estimated maximum required downlink data rate is 100 kbps.

The required data-rate on the uplink ($DR_{UL}$) is determined by the desired sampling rate ($SR$) and accuracy of LFP recording (i.e. ADC size on the recording) on each optrode as well as the number of the recording sites ($N_{rec}$) and optrodes ($N_{optrode}$). A real-time communication of the recorded LFP data to the chest unit is highly desirable in order to avoid the need for a memory block on each optrode. Therefore $DR_{UL}$ at which the chest unit receives the LFP data from the brain implant is:

\begin{equation*} DR_{UL}= N_{optrode}\times N_{rec} \times SR \times {DP_{UL}} \end{equation*}

where $DP_{UL}$ is the uplink data packet size and is described in Section V-D.

The target number of recording sites and LEDs are determined based on the requirement of recording and stimulation spatial resolution, which is beyond the scope of this paper. However, considering both power limitation due to temperature rise in the tissue (maximum allowed $0.5^{o}C$) [16] and implementation effort, a platform consisting of 16 optrodes has been selected as the target system, with key parameters listed in Table I.

**TABLE I:** Parameters for the Implant System

Parameter	Value	Parameter	Value
$N_{optrode}$	up to 16	Sampling rate	0.5–1 kS/s
$N_{rec\_{}sites}$ ^*^	4	$N_{LED}$ ^*^	4
UL packet	26-bit†	DL packet	26-bit†
$DR_{up,max}$	1.6 Mbps	$DR_{down}$	100 kbps

^*^ Per optrode,

† Drawn in Section V-D

**Fig. 2.**
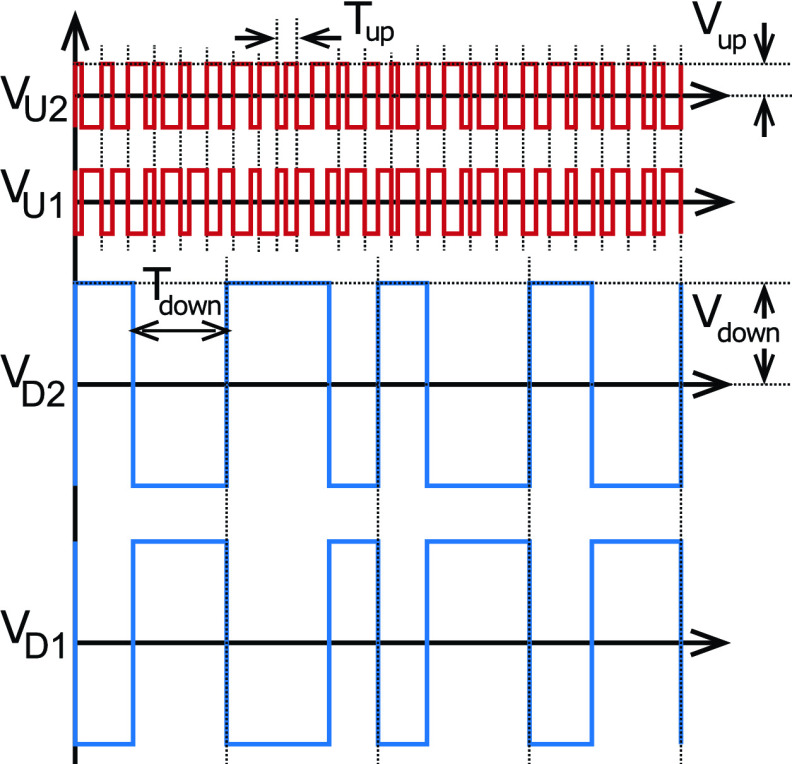
Ideal voltage waveforms on the 4-wires (when the wire impedance and the wire-to-wire conductance of wires are both zero). Subscripts D1 and D2 are for downlink, U1 and U2 are uplink wires.

Each optrode contains 3 recording sites and 8 stimulation channels (i.e. mini-LEDs). Although certain recording channels or even entire optrodes can be disconnected optionally from the system, a data rate of 1.6 Mbps, from Eq. 1, is required to cover the maximum possible bandwidth for the uplink. An example of waveforms on the lead wires can be found in Fig. 2. The subscript D1 and D2 refer to the pair of wires used for downlink, and U1 and U2 refer to the wires used for uplink.

## III. Communication Channel – 4-Wire Lead

**Fig. 3.**
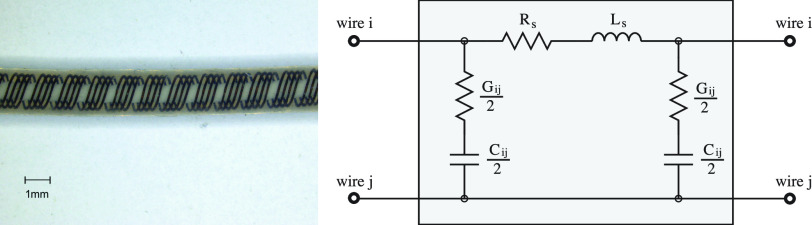
Photograph of the Cooper cable and the lumped $\Pi $ model used in lead characterization.

An implantable lead was chosen which has been approved under both the FDA and EU regulations: Cooper cable [19], shown in Fig. 3. This consists of four wires arranged as equally-spaced coaxial helices. The Platinum-Iridium wires are enameled with polyimide and the helices are embedded in silicone rubber.

This particular design is chosen for mechanical reasons: the helical configuration makes the cable extremely flexible, allowing stretching, twisting and bending without damaging the cable and without significantly altering the distance between the wires. The main drawback of a coil structure, however, is the increase in electrical resistance per unit length, which increases the power dissipation in the wires. The cable also exhibits unmatched characteristics across the different conductors.

### A. Lead Characterization

Electrical characterization of the lead was performed on a 77 cm long 4-wire Cooper cable provided by Finetech Medical Ltd. The measurements were performed with Keysight E4980A/AL Precision LCR Meter in a frequency range including frequencies employed in uplink (100 kHz) and downlink (up to 1.6 MHz), respectively.

A 2-port network characterization of the lead is performed in air between each pair of wires within the 4-wire lead. All 6 different pair combinations were measured. During each measurement the conductors not in use were kept open on both ends i.e. left floating.

A lumped element $\Pi $ model is defined (Fig. 3) to represent the line parameters. A lumped model is chosen over a distributed model because the required length of the implanted cable, $l_{cable}$ is significantly smaller than the wavelength of the signal. The line parameters (valid for a 77 cm Cooper cable) are therefore derived from the discrete element model by measuring 3 impedances: the equivalent impedance Zi for each single wire, the short-circuit impedance Zsc and the open-circuit impedance Zoc for each combination of two wires (6 different two-port network combinations). Zsc is measured on one end of a single two-port network when at the other end the two wires are shorted with each other (the other two wires are left open and floating), whereas Zoc is obtained by measuring one end and leaving the two wires at the other end open (the remaining two wires are left open and floating).

The series resistance $R_{s}$ and inductance $L_{s}$ are derived from the real and imaginary parts of $Z_{i}$. The dielectric capacitance $C_{ij}$ and the dielectric conductance $G_{ij}$ between each pair of wires are extracted from the measured $Z_{sc}$ and $Z_{oc}$ using Eqns. 2 and 3.

\begin{align*} Zsc_{ij}=&\left({ \frac {G_{ij}}{2} - {\frac {j}{\pi f C_{ij}}} }\right) \parallel \big ({ R_{s} + j 2 \pi f L_{s}} \big) \\ Zoc_{ij}=&\left({ \frac {G_{ij}}{2} \!-\! \frac {j}{\pi f C_{ij}} }\right) \parallel \left({ R_{s} \!+\! j 2 \pi f L_{s} \!+\! \frac {G_{ij}}{2} \!-\! \frac {j}{\pi f C_{ij}} }\right)\notag \\ {}\end{align*}

The model parameters are summarized in Table II. For each parameter both the average value and its variation among the wire(s) within the same category (i.e. adjacent, non-adjacent) are given. For example, the $R_{s}$ and $L_{s}$ of each wire are within 1 % of the average resistance and inductance of all wires and remain so by increasing the frequency to $f_{up}$. The 2-port related parameters instead depend on the choice of the two wires: the adjacent wires have almost 25 % higher $C_{ij}$ compared to the non-adjacent ones. The extracted parameters are almost frequency-independent except for $G_{ij}$. The frequency-dependency of $G_{ij}$ and $C_{ij}$ may be attributed to the approximation made in the lumped model compared to a distributed model.

**TABLE II:** Measured Lumped $\Pi $ Model Parameters of 77 cm Cooper Cable in Air

Parameter	Unit	100 kHz	1.6 MHz
$R_{s}$	$\Omega $	313± 3	310± 3
$L_{s} $	$\mu \text{H}$	3.59± 0.05	3.59± 0.05
$C_{ij}$, adjacent^*^	pF	118±2.4	122± 2.5
$G_{ij}$, adjacent	mS	19.2± 0.5	29.3± 1.2
$C_{ij}$, non-adjacent	pF	89± 1.3	92±1.5
$G_{ij}$, non-adjacent	mS	22.5± 0.7	27.3±0.4

^*^ Adjacent wires: W1-W2,W2-W3,W3-W4.

### B. Capacitance Equalization Network

The lead parameters that affect the efficiency of the power and data transmission are *(i)* series resistance, $R_{s}$, that causes the thermal energy loss and the $R_{s}I$ drop; *(ii)* the parallel capacitance, $C_{ij}$, causes dynamic power loss between each pair of wires. To minimize the dynamic power loss on the lead, we choose a non-adjacent pair of wires for power/downlink transmission (i.e. W1 and W4; Because $C_{14}$ is slightly smaller than $C_{13}$ and $C_{24}$).

On the other hand, any asymmetry between the $C{ij}$ from the uplink wire, U1 (or U2), to the two downlink wires, D1 and D2, induces a square-wave voltage at the frequency of $f_{down}$ on U1 (and U2). The peak-to-peak of the induced voltage (measured differentially between U1 and U2) is as high as 4 V depending on which pair of wires are chosen for uplink/downlink. For example, when W1 and W4 are used for downlink the differential peak-to-peak voltage induced on W2 and W3 (i.e. uplink wires) is 2.1 V. To mitigate this, a capacitive equalization network is added such that the total capacitance from U1 (or U2) to D1 becomes equal to the total capacitance between U1 (or U2) to D2. The optimum equalization (i.e. maximum reduction of the induced voltage on U1 and U2) was achieved in the experiments when two similar sub-networks were placed at either ends of the lead instead of one network on one end. This could be attributed to the use of the relatively long lead where the measured induced voltage on the head side of U1 and U2 is in fact the collective effect of interference that happened along the whole length of the wire. Therefore, it can be fully equalized using a distributed (here two) equalization network rather than a single lumped network. Each sub-network includes two capacitors, $C_{CE}$. Adding an external capacitor between a single pair of wires affects the total capacitance between all other pairs as well. The minimum $C_{CE}$ to equalize the capacitances between the uplink and downlink wires was found from impedance measurements to be 27 pF. By adding the equalization network, the average of the total capacitance between U1 and U2 to D1 and D2 increases to 147 pF while their variation form the average value remains at ±2 pF; The capacitance between the downlink wires, D1 and D2, increases to 121 pF.

## IV. Power Transmission Efficiency

The amplitude of the voltage at the chest, $V_{down}$ to drive the downlink and power is dynamically adjusted by the chest unit to:

\begin{equation*} V_{down}= V_{optrode}+ V_{drop,rect}+ R_{s} I_{avg} \end{equation*}

where $V_{optrode}$ is the output of the rectifier and needs to have an average of 5 V for LED stimulation through dedicated circuits [17], $V_{drop,rect}$ is the voltage drop across the rectifier (measured 1.15 V). $I_{avg}$ is the average of the absolute current taken from the chest implant on $W_{D1}$ (or $W_{D2}$) during stimulation,

\begin{equation*} I_{avg}= N_{optrode} I_{op,nrm}+ I_{op,stim} \end{equation*}

where $I_{op,nrm}$ is the current consumption of each optrode when not stimulating (about 0.2 mA), and $I_{op,stim}$ is the peak stimulation (LED bias) current (1.5–2.3 mA depending on stimulation intensity). The stimulation phase is such that only and always one LED per the entire head unit is on at any given time, though the stimulating optrode may change during the stimulation phase.

Since the voltage on each differential wire is square waveform, the average transmitted power from the chest, disregarding the rise/fall time of the square-wave, is:

\begin{equation*} P_{chest,avg}=V_{down}I_{avg} \end{equation*}

The total dynamic power loss on the lead, $P_{chest,dyn}$, is due to the charge and discharge of the $C_{ij}$ capacitances between the wires. This is formulated in Eqn. 7 where $\alpha _{data,down}$ is a scaling factor between 0.5 and 1 that depends on the number of transitions in the downlink data: it is 1 for a data stuck at 0, 1, or during channel idle mode (i.e. when no data is sent on downlink), and is 0.5 for an alternating downlink data bit pattern as when this is Manchester encoded (i.e. data XOR clock) any transition in data is encoded into a fixed level.

When the uplink is inactive the voltages at the uplink wires, U1 and U2, within the DC-blocking capacitors remain at the average voltage between downlink wires, D1 and D2. Moreover, with the addition of the capacitance equalization network, the total capacitance between all pairs except $W_{D1}$ and $W_{D2}$ is equal to the $C_{UD,tot}$. Therefore, Eqn. 7 can be rewritten to Eqn 8 assuming the voltage on each wire is constant throughout its length (i.e. disregarding $R_{s}I$ drop) and the downlink channel is idle.

\begin{align*} P_{chest,dyn}=&\alpha _{data,down} f_{down}\notag \\&\times \Sigma C_{ij}V^{2}_{ij},\quad i,j=1~to~4;,~ i\neq j \\ P_{chest,dyn}\simeq&f_{down}V^{2}_{down}(C_{D1D2,tot}+ C_{UD,tot}) \end{align*}

The power transmission efficiency (PTE) is defined as the ratio of the delivered DC power to the load (i.e. total load of rectifiers on all optrodes) to the total power transmitted by the chest implant. This is calculated in Eqn. 9.

\begin{align*} PTE=&\frac {P_{load,tot}}{P_{chest,avg}+P_{chest,dyn}}\notag \\ P_{load,tot}=&V_{optrode}I_{avg} \end{align*}

**Fig. 4.**
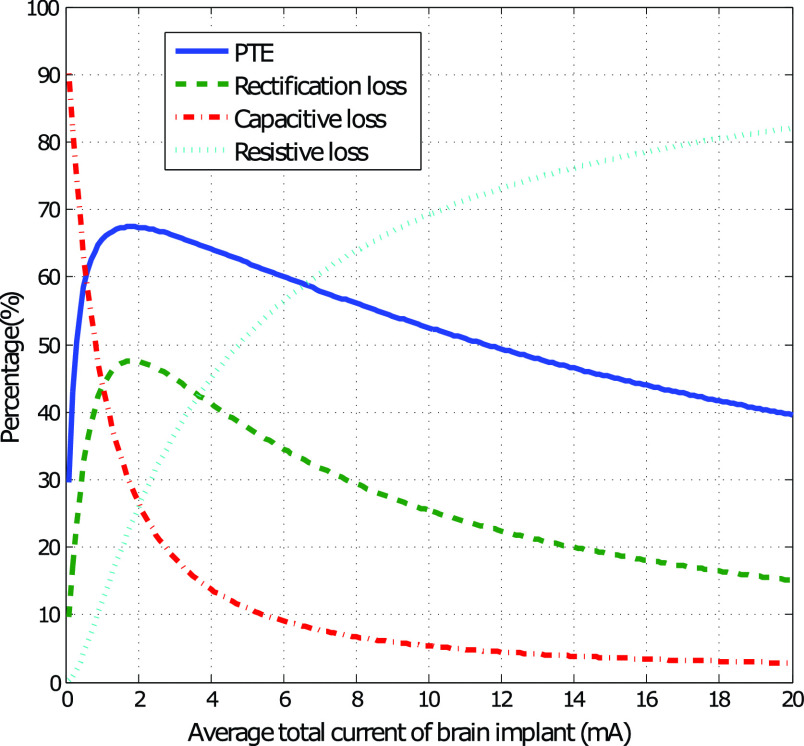
The simulated power transmission efficiency versus the average current consumed on the multi-optrode brain unit. Also shown is percentage contribution of the resistive and capacitive loss on the 4-wire lead as well as the power loss due to voltage drop on the full-wave rectifier ($V_{drop,rect}$ reported in Table III).

The PTE is plotted versus $I_{avg}$ in Fig. 4 using a 77 cm Cooper cable (simulation parameters taken from Table II). The percentage contribution of the three main power loss sources (resistive, capacitive and the voltage rectifier) are plotted for comparison. The maximum achievable PTE is 67.7% at a current of 1.9 mA. The drop in efficiency at lower currents is due to the dominant effect of the dynamic power loss, while at higher currents the effect of the thermal energy loss on the lead is dominant.

By activating the uplink on an optrode the dynamic power loss, $P_{chest,dyn}$, increases by:

\begin{equation*} P_{up,loss,dyn}\simeq 4\alpha _{act} N_{optrode} \alpha _{data,up}f_{up} C_{ij,max} V^{2}_{up} \end{equation*}

where $V_{up}$ is the amplitude of the received uplink waveform, $\alpha _{act}$ is the activity ratio of each optrode within TDMA and has a maximum of $1/N_{optrode}$, $\alpha _{data}$ is between 0.5 and 1 and depends on the number of transitions in uplink data. Although this power is consumed by the optrodes and is therefore in the nominator of the PTE, it reduces the total power efficiency of the system.

## V. Circuit Implementation

The system presented herein is monolithically integrated within the optrode to interface between the shared 4-wire lead and the optrode core [18]. This interface receives from the downlink (i.e., recovers/provides a stable DC power supply, generates an upscaled clock at 1.6 MHz and decodes control data), and sends to the uplink (encoding the recorded data). The chest implant requires a complementary interface to drive the downlink (encoding control data with power carrier) and receive/decode the uplink data.

**Fig. 5.**
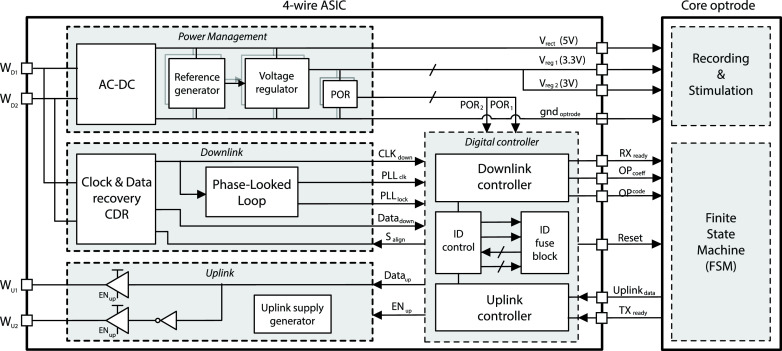
Top-level system architecture for the 4-wire interface ASIC.

The top level architecture of the 4-wire interface system is shown in Fig. 5, divided into three main blocks: *(i)* Power management; *(ii)* Downlink; *(iii)* Uplink; and *(iv)* digital controller.

### A. Power Management

This consists of a full-wave voltage rectifier generating the required 5 V DC-supply for driving the LEDs (within each optrode) that can operate from an unregulated supply. A voltage regulator generates the 3.3 V regulated supply for powering the optrode core circuits. This is gated by a *power-on-reset* (POR) circuit (adapted from the AMS C35B4 A_CELLS library) such that the regulator output is disconnected from the load during startup, and in the event of large voltage drops. To separate the supply voltage of the analog and digital circuits within the optrode, a duplicate of the reference generator and voltage regulator generates a second supply voltage at 3 V.

#### 1) Full-Wave Voltage Rectifier:

The system uses square waveforms for power transmission. Key advantages here (over sinusoidal) are: *(i)* this makes it possible to use an on-chip smoothing capacitor to suppress the output ripple; *(ii)* it is easier to generate, thus reducing complexity of the chest implant.

**Fig. 6.**
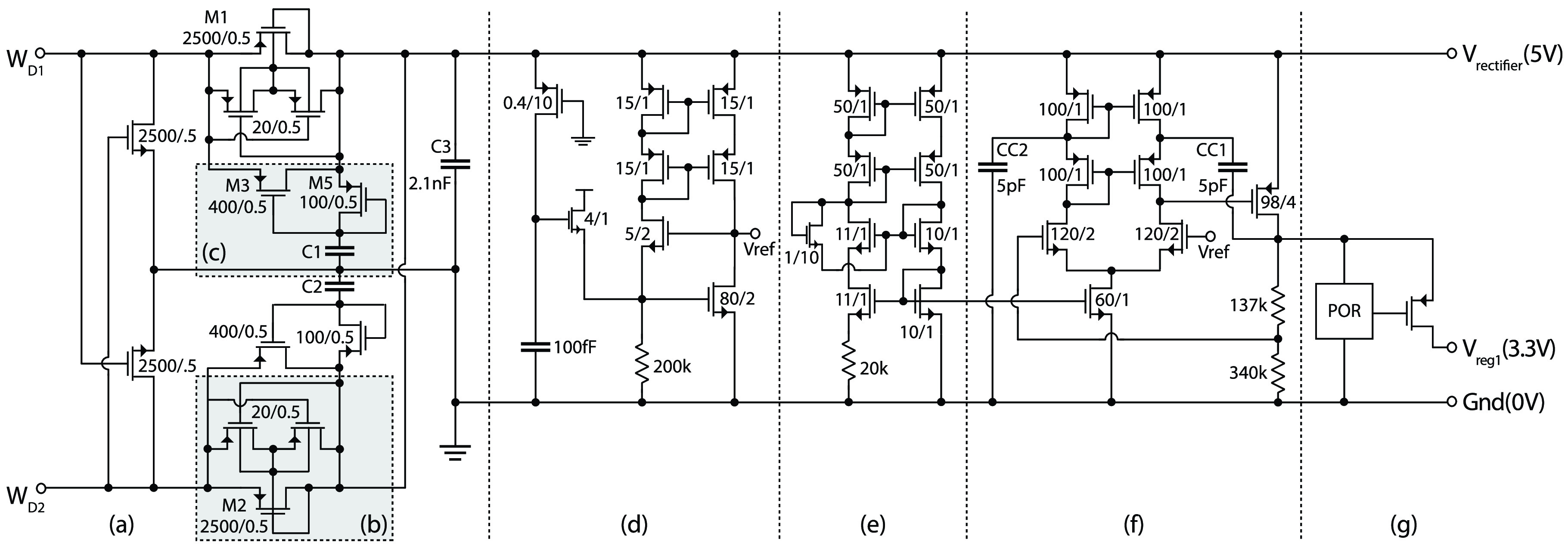
Circuit schematic of the power management unit on the 4-wire interface ASIC showing: (a) full-wave voltage rectifier with (b) bulk biasing, and (c) charge storage techniques; (d) reference voltage generator with start-up circuit; (e) bias voltage generator for (f) voltage regulator; (g) connections to the POR circuit for power gating during start-up.

The implemented circuit uses a passive rectifier (Fig. 6). This is preferable because: *(i)* An active rectifier would require extremely fast (thus power hungry) comparators to recover the square-wave; *(ii)* the risk of instability on the fast transitions of the square-wave. The passive rectifier circuit is based on the topology used in [21], consisting of a diode-connected PMOS pair and cross-coupled NMOS pair.

The PMOS bulks are biased at the highest potential using a dynamic body biasing technique. Moreover, since $V_{th}$ is quite high at 5 V devices the rectifier would have a large voltage drop which limits the power transmission efficiency. In order to reduce the voltage drop, a charge-storage technique is used [22] by the addition of $M_{3}$, $M_{5}$ and $C_{1}$.

The rectifier output is smoothed using a 2.1 nF on-chip capacitor that is formed by stacking MIM (0.55 fF/$\mu \text{m}^{2}$; formed between Metal 2 and Metal 3 layers) and MOS capacitors (2.55 fF/$\mu \text{m}^{2}$; formed between Poly and Metal 1 layers) to increase the total capacitance density. Simulations indicate that the rectifier can source up to 2.5 mA with peak-to-peak voltage ripple of less than 0.5 V when transient time of the input square-wave is within 5% of its period.

#### 2) Voltage Regulator:

This is designed to provide a stable 3.3 V DC voltage from the unregulated 5 V supply provided by the rectifier. A low-dropout (LDO) regulator was chosen over a DC-DC converter because the efficiency enhancement provided by DC-DC converter is not significant from 5 V to 3.3 V. Moreover, LDO provides robust start-up operation without generating high frequency interference and extra internal passive devices.

The circuit schematic of the voltage regulator is shown in Fig. 6. This is based on a telescopic amplifier topology driving a pass-transistor that provides the negative feedback, using Miller compensation to eliminate the forward zero by connecting the Miller capacitor to the cascode device. A second compensation capacitor ($CC_{2}$) is used to improve the symmetry within the telescopic amplifier, thus improving the *Power Supply Rejection Ratio* (PSRR). More specifically, the role of $CC_{2}$ is to move the two uncorrelated zeros at the cascode node to a pair of complex zeros in the left half-plane. A transient analysis here demonstrate better stability over wider range of switching load.

The regulator bias is generated on-chip using a beta-multiplier circuit. The voltage reference used has been adopted from [23] to provide a high PSRR. Here, the negative feedback around the NMOS devices ensures that output voltage ($V_{ref}$) remains constant over a wide range of supply voltage. This operates, for example, as follows: an increase in supply voltage will cause the current through the resistor to increase, in turn increasing the current in the output branch, that will decrease the output voltage.

### B. Full-Duplex Communication – Downlink

This consists of two key blocks for recovering the data and clock from $W_{D1}$ and $W_{D2}$: *(i) Clock and Data Recovery* (CDR) converts the signal from 5 V differential, to 3.3 V single-ended, and recovers the data and downlink clock ($clk_{down}$@100 kHz); *(ii)* a frequency synthesizer Phase Locked loop (PLL) generates a higher frequency clock ($clk_{up}$@1.6 MHz) from $clk_{down}$ to drive the uplink as well as the optrode core logic.

#### 1) Clock and Data Recovery (CDR):

**Fig. 7.**
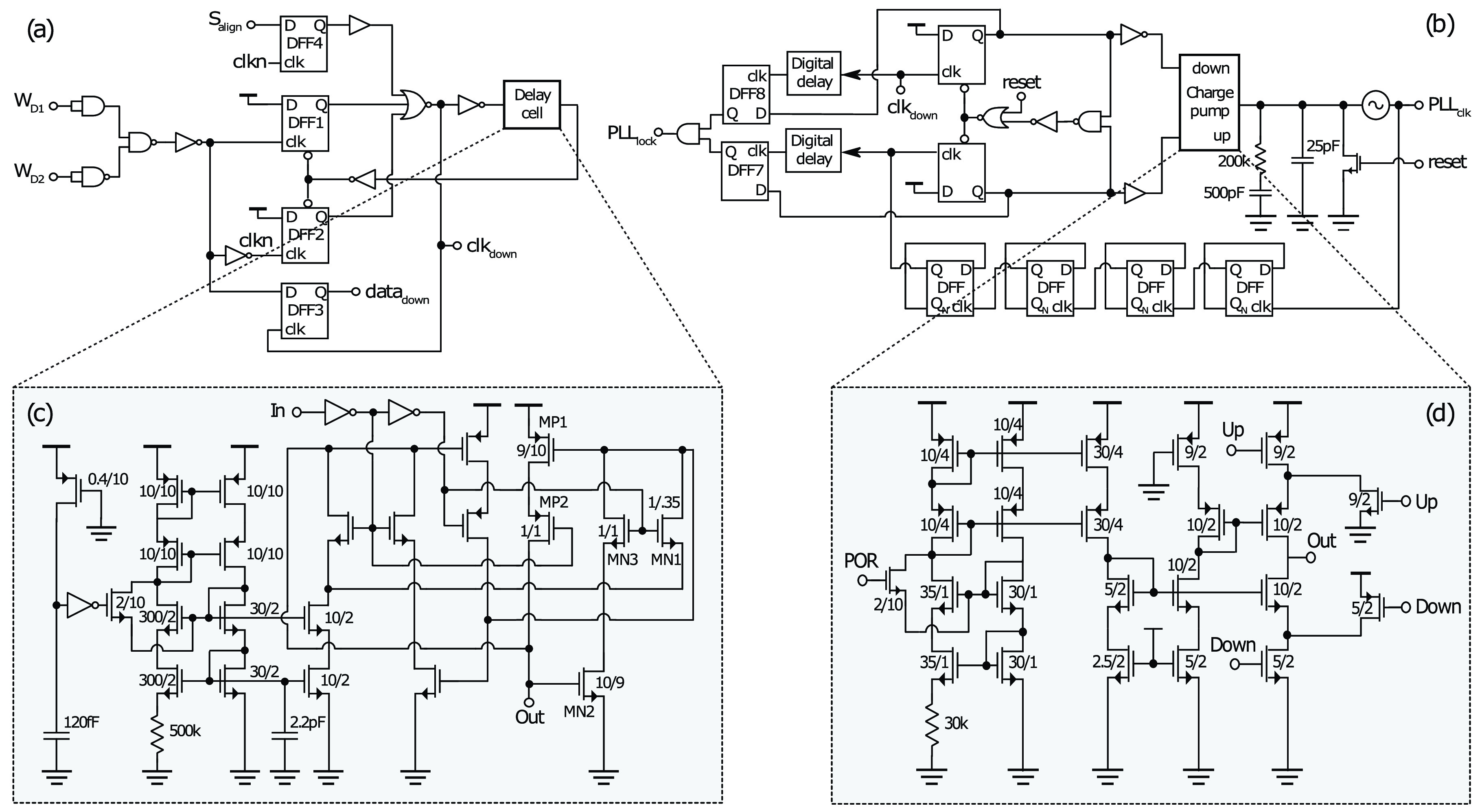
Downlink circuit schematics showing: (a) clock and data recovery (CDR); (b) phase-locked loop; (c) delay cell used in CDR; (d) charge pump circuit used in the phase-locked loop.

The CDR circuit is shown in Fig. 7–(a). The differential voltage between at $W_{D1}$ and $W_{D2}$ is fed into a non-hysteretic comparator consisting of two NAND and one AND gates. An inverter (with thick oxide transistors) powered from the 3.3 V supply then shifts the signal level from 5 V to 3.3 V. An edge detector, consisting of two edge-triggered D-type flip-flop (DFF1 and DFF2) and a delay cell detects both the rising and falling edges of the signal; The reset of DFF1 and DFF2 is driven by the delayed version of the pulses generated on either of the DFFs. A delay of $T_{d}$ within $T_{down}/2$ and $T_{down}$ (the period of the downlink clock) is provided through the delay cell to recover the clock correctly.

A thyristor-based delay circuit generates the delay of $T_{d}$ [24]. The schematic of the delay circuit is shown in Fig. 7–(c) and has two half-circuits, activated at different edges of the input signal. Each half-circuit functions as the others precharge circuit so the half-circuit is turned off and precharged before the next transition.

When input of the delay cell has a low-to-high transition, the gate of $MP_{1}$ discharges with a constant current, $I_{ref}$ through $MN_{1}$. Once the gate-source voltage of $MP_{1}$ reaches the threshold voltage it turns on and charges up the $MN_{2}$ gate through a positive feedback.

The positive feedback mechanism provides a quick flipping of the state and reduces the dynamic power consumption as no current flows directly from $V_{DD}$ to ground. The switch transistors $MN_{3}$ and $MP_{2}$ are used to prevent shunt current.

The reference current, $I_{ref}$ is generated on-chip by a beta-multiplier circuit. To ensure that $T_{d}$ remains within $T_{down}/2$ and $T_{down}$ the resistor and transistors within the current reference generator are carefully sized and a smoothing capacitor (2.2 pF) is added to reduce any switching feedthrough. Robust data and clock recovery has been confirmed through corner simulations across a 10 % supply variation, 30–80°C temperature range, and through typical, fast and slow corners.

The clock recovery in the CDR circuit is symmetrical with respect to $W_{D1}$ and $W_{D2}$, meaning the clock may align to either $W_{D1}$ or $W_{D2}$ depending on the start-up condition. To ensure the recovered $clk_{down}$ is always aligned to $W_{D1}$ (and not $W_{D2}$), a one-off alignment-correction method is embedded within the CDR through DFF4 driven by an active-high signal $S_{align}$. $S_{align}$ is activated after start-up by the digital controller for a total time of at least $T_{Dl}$. The following delay cell (DLY) produces a non-critical delay of 2 ns with digital gates to ensure correct transition time with respect to other DFFs.

#### 2) Phase-Locked Loop:

This is shown in Fig. 7–(b), consisting of a phase and frequency detector, charge pump, voltage-controlled oscillator (VCO) and a low-pass filter. The charge pump is adopted from [25], supplying a nominal current of $1~\mu \text{A}$ to the low-pass filter. Cascode stages and long channel transistors are used in order to decrease channel length modulation and mismatch effects as well as to achieve higher output resistance. The VCO is implemented using a five-stage current-starved ring oscillator followed by a level shifter and output buffer.

A lock-detector is embedded within the PLL, through DFF_7_, DFF_8_ and two digital delay cells (from AMS digital standard cell library), to acknowledge lock of phase in the PLL. The lock signal is used to reset the optrode core circuits together with the POR signal. The lock-detector inserts the lock signal when PLL has reached a steady-state condition. This is when activity period of the charge-pump is smaller than the delay created by the delay-cells (i.e. 300 ns).

### C. Full-Duplex Communication – Uplink

The Manchester encoding is implemented by an XOR gate which combines the uplink data with uplink clock in the digital controller and sends encoded $data_{up}$ through two tri-state buffers on the wires. The uplink clock is decided by the FSM and can be as high as 1.6 MHz as generated by the PLL. Two tristate buffers are used to drive the encoded data to the channel when the digital controller asserts an $EN_{up}$. To reduce the power consumption of the uplink, the supply voltage of the tristate buffers is reduced to $V_{up}$. A buffer amplifier powered by the voltage rectifier is designed to generate $V_{up}$ from one of existing reference voltages in the power management circuits (i.e. $V_{reg1}$,$V_{reg2}$,or $V_{ref}$).

Key circuit specifications that have been determined through simulations are summarized in Table III.

**TABLE III:** Circuit Specifications

Circuit	Parameter	Value	Unit
Rectifier	$\text{V}_{out}$	≈5	V
	$\text{I}_{out,max}$	2.5	mA
	$\text{V}_{drop,rect}$	1.15	V
Reference generator	$\text{I}_{DD}$	8	$\mu \text{A}$
	$\text{V}_{ref}$	2.09	V
	PSRR@0.2 MHz	65	dB
Voltage regulator	PSRR@1 kHz	76	dB
	PSRR@0.2 MHz	46	dB
	$\text{I}_{DD}$	21	$\mu \text{A}$
	line regulations	0.2	mV/V
PLL	Lock-in range	33–200	kHz
	$\text{I}_{DD,avg}$	10.5	$\mu \text{A}$
	start-up time	$70\times T_{clk,in}$	
	rms jitter	70	ps
CDR	$\text{I}_{DD,avg}$	1.2	$\mu \text{A}$
	$T_{d}$	7.5± 2	$\mu \text{s}$
Uplink	$\text{I}_{DD,avg}$	21.5	$\mu \text{A}$
Total	$\text{I}_{DD,avg}$	91.2	$\mu \text{A}$
	area	1.5	mm^2^

### D. Digital Controller

A dedicated digital controller was designed that consists of the following main blocks: *(i)* ID block and corresponding driver; *(ii)* downlink receiver, identifies valid commands and following operation coefficients from the recovered data; It also provides the signal $S_{align}$ for the CDR. *(iii)* uplink transmitter prepares the uplink data packet and generates the signal $EN_{up}$ to activate uplink transmission. It also generates the Manchester encoded $Data_{up}$ from uplink data and clock using an XOR gate. The controller resets the core optrode by generating a synchronized reset based on the POR and $PLL_{lock}$ signals.

Each optrode has unique identifier (ID) that is defined using a one-time programmable (OTP) fuse. The ID is used in downlink, for commands to addressed a particular optrode, and in uplink, to sign the data packet. In this system, the AMS IP block P2RAM3V is used as the ID block on each optrode [26]. The block has a 32-bit fuse based one-time-programmables memory and a corresponding register. Only 6 bits are used as ID bits to accommodate for a maximum of 63 optrodes (0 reserved for commands). The corresponding controller is designed such that the IP block can be used in either the debug mode (i.e. only the registers are used) or fuse mode (i.e. the fuse memory can be updated and used as address code). To further reduce the size and power of ID block, a dedicated low power physically unclonable function generator can be used, such as in [27] and [28].

**Fig. 8.**
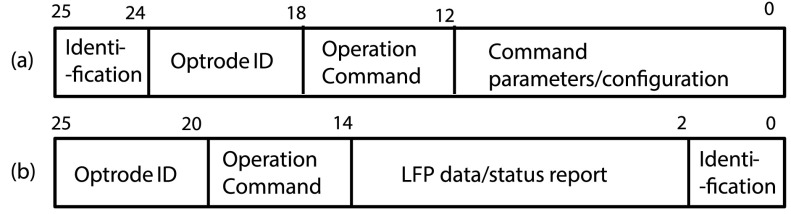
Data Packet for: (a) downlink; (b) uplink.

The data packet structure for the downlink and uplink are shown in Fig. 8–(a) and 8–(b). A 2-bit packet identification pattern is used at the beginning to signal the arrival of a data packet, as well as to enable validation of the packet on the receiver side. This is followed by a 6-bit optrode ID, to indicate the target optrode in downlink command or the origin of the uplink data. The downlink packet also includes a 6-bit operation command followed by 12-bit operation coefficient. The uplink sends the executed 6-bit command followed by a 12-bit data.

## VI. Fabricated Device

**Fig. 9.**
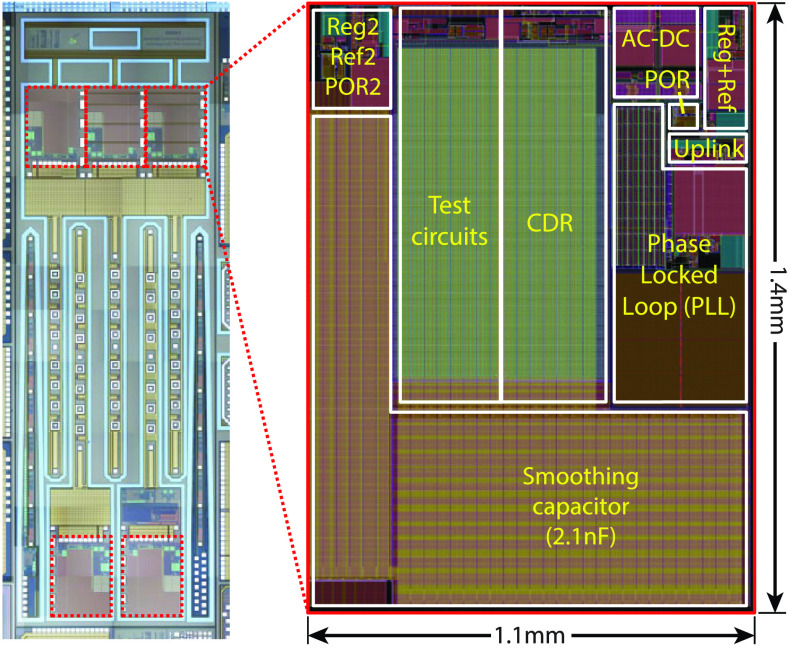
Fabricated 4-wire system ASIC implemented in $0.35~\mu \text{m}$ CMOS technology. Shown are: (a) microphotograph of optrode system (5 optrodes shown); (b) layout and annotated floorplan of the 4-wire interface core.

The 4-wire ASIC has been implemented in AMS 0.35 $\mu \text{m}$ 2P4M CMOS technology (H35B4S3) occupying a silicon footprint of 1.5 mm^2^. Thick oxide transistors have been used for all circuits that interact with $W_{D1}$ and $W_{D2}$. The digital controller is synthesized together with the main optrode FSM. The power management and full-duplex communication circuits have been configured and fabricated in two different form factors: *(i)* as a complete system embedded within the optrode to be used in the end-application, with only access points being connections to the 4-wire lead; and *(ii)* as a standalone test circuit, with the CDR, PLL and power management unit are accessible for testing purposes. Inverting voltage buffers were used on the digital outputs $data_{down}$, $clk_{down}$, $PLL_{lock}$, and $PLL_{clk}$. The buffers are powered through the same supply voltages powering the CDR and PLL. The IO ring was connected to the rectifier output. The microphotograph and layout with annotated floorplan are shown in Fig. 9.

The 4-wire system ASIC is connected to the core optrode on the complete system, providing the power supply and establishing data links. The core optrode system consists of: *(i)* digital controller to control the front-end recording and stimulation; *(ii)* data converters; *(iii)* neural amplifiers; *(iv)* LED drivers. Recording and LED sites are distributed along the shaft of the implants.

**Fig. 10.**
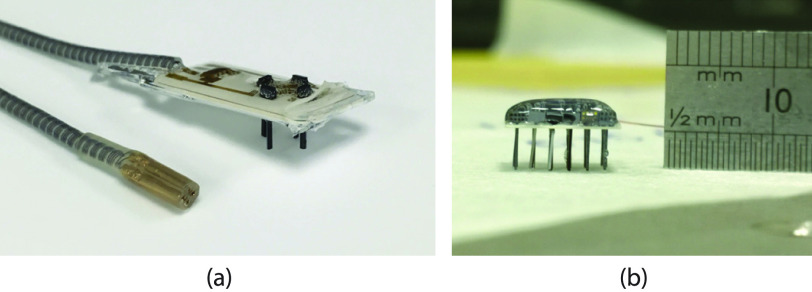
Preliminary prototypes for the multi-optrode units. Shown are: (a) four optrode assembly mounted on ceramic base plate connected to the 4-wire Cooper cable (shown with Craggs connector); (b) 15 optrode assembly (arranged in $5\times 3$ array) with silicone encapsulation. 4-wire interface ASIC (described herein) implemented in the ‘head’ of each optrode probe.

At the end of the brain implant, the lead is connected to a ceramic baseplate that carries the silicon optrodes. The DC-blocking capacitors as well as the capacitance equalization network on the brain side of the lead are also placed on the baseplate and electrically connected through printed gold tracks. Fig. 10 shows a photograph of the encapsulated baseplate with multiple optrodes. Details of the brain implant will be described in forthcoming papers.

## VII. Measured Results

**Fig. 11.**
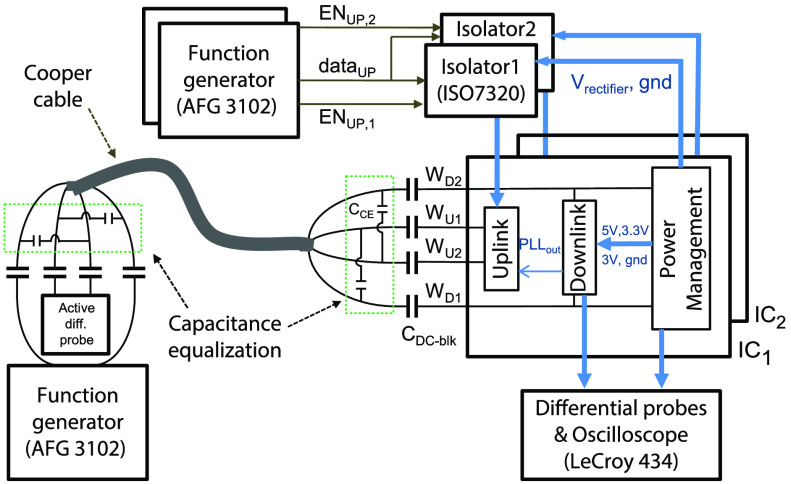
Experimental setup used for device measurements.

The 4-wire ASIC was tested under various conditions using a 2-channel function generator to provide the power/downlink. The lead presented in Section III was connected between the function generator and the IC(s) through two $1.5~\mu \text{F}$ DC-blocking capacitors per wire, placed at either ends of the wires. While in theory one capacitor per wire is enough to decouple the chest and head units, in this work two DC-blocking capacitors per wire are used for safety reasons. This is to ensure that in case of a breakage on the lead and/or the DC-coupling capacitors on the chest side, the head unit is still decoupled from the rest of the system and any DC current is prohibited. The wires W1 and W4 were used for downlink, and W2 and W3 for uplink. The capacitance equalization network was added at both ends of the lead as discussed in Section III-B to eliminate the interference of downlink on uplink. The measurement setup is sketched in Fig. 11. All voltages were measured differentially either between two wires within the lead or with respect to the local ground at the output of the rectifier. The rise/fall time of the square-waves on the function generator were set to 50 ns and the amplitude was tuned (according to Eqn. 4) such that the voltage rectifier outputs an average unregulated voltage of 5 V.

The power transmission efficiency was measured using one 4-wire ASIC connected to the function generator as described above. A small resistor ($100~\Omega $) was added in series with W1 between the function generator and the ac-coupling capacitor to measure the instantaneous current taken from the function generator. The PTE was then calculated by dividing the average output power of the rectifier to the average of the absolute instantaneous power at the output of the function generator. The measured PTE is 38% at load current (i.e. $I_{avg}$ in Eqn. 5) of 0.25 mA and increases by increasing the current with a peak of 66% at 1.4 mA. Further increasing the current results in a gradual drop in PTE down to 55% at 5.9 mA. The discrepancy between measurements and simulation results could be attributed to limited accuracy and potential error in the measurement setup as well as disregarding the inductance of the cooper cable in the simulations.

The CDR and PLL were powered through the two on-chip regulators, Vreg_1_ and Vreg_2_, respectively. A load of 120 $\text{k}\Omega $ ($27~\mu \text{A}$) and 100 pF was added to Vreg_1_ to model the load and parasitics of the core circuits within the optrode. A load of 50 $\text{k}\Omega $ ($60~\mu \text{A}$) and 220 pF was added to Vreg_2_ for the same reason. Therefore, the total current taken from the chest implant is $178~\mu \text{A}$ per optrode including the standby current consumption of the 4-wire circuit itself.

### A. Downlink and Power Management

**Fig. 12.**
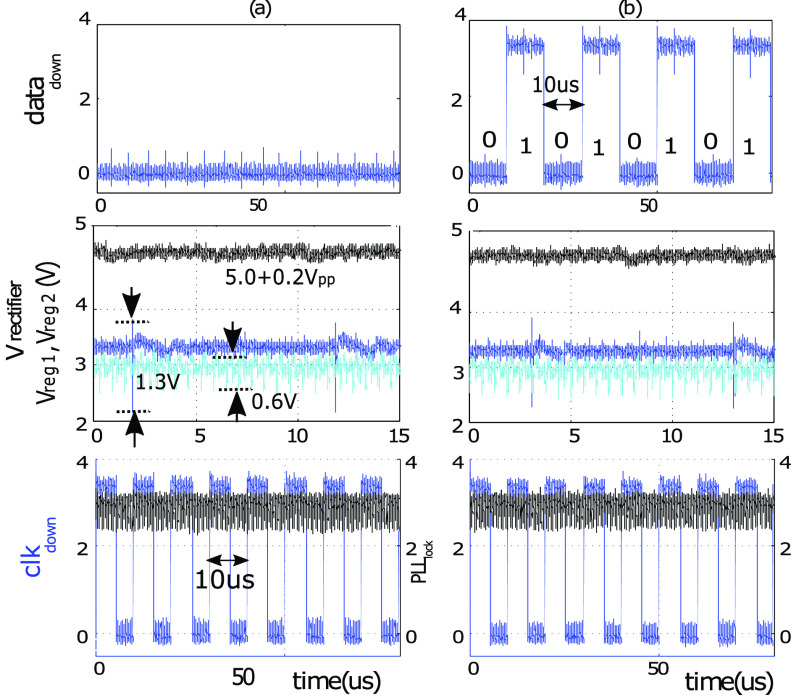
Measured waveforms at the power management and downlink blocks of the 4-wire ASIC when (a) $\text{V}_{down}$ at 100 kHz was sent through $\text{W}_{D1}$ and $\text{W}_{D2}$ to the 4-wire interface; (b) the frequency of $\text{V}_{down}$ was changed to 50 kHz representing alternating data pattern.

The downlink and power management circuits were tested under two different conditions and results are shown in Fig. 12. *(i)* a single IC was driven by a $V_{down}$ (square-wave at 100 kHz frequency); The recovered $data_{down}$ is stuck at zero while $clk_{down}$ successfully recovers the 100 kHz clock. The PLL, driven by $clk{down}$, locks to the correct frequency (i.e. the signal $PLL_{lock}$ remains high). *(ii)* The frequency of $V_{down}$ is changed to 50 kHz; This represents a downlink data with alternating bit pattern in Manchester encoded data, and is correctly recovered by the CDR while at the same time the PLL remains in the locked condition.

In both measurements the ripple on the rectified 5 V is 200 mV$_{pp}$. The transient voltage spikes on Vreg_1_ (connected to CDR) are up to 1.3 $\text{V}_{pp}$ with a duration of almost 100 ns and happen during each voltage transient on $clk_{down}$. The Vreg_2_ (connected to PLL) has spikes with upto 600 mV$_{pp}$ at 1.6 MHz frequency due to switching activities within PLL. The spikes decays within almost 100 ns.

### B. Uplink on Two-ASIC System

The uplink communication was tested using two 4-wire ICs connected in parallel to the 4-wire lead. To study the effect of using different uplink clock frequencies, two experiments were run. In the first experiment the $PLL_{out}$ was used to drive $data_{UP}$ at 1.6 MHz. In the second a function generator was used to provide a different clock. In both experiments the voltage $V_{ref}$ on the uplink circuit was tied to Vreg_2_ at 3 V.

**Fig. 13.**
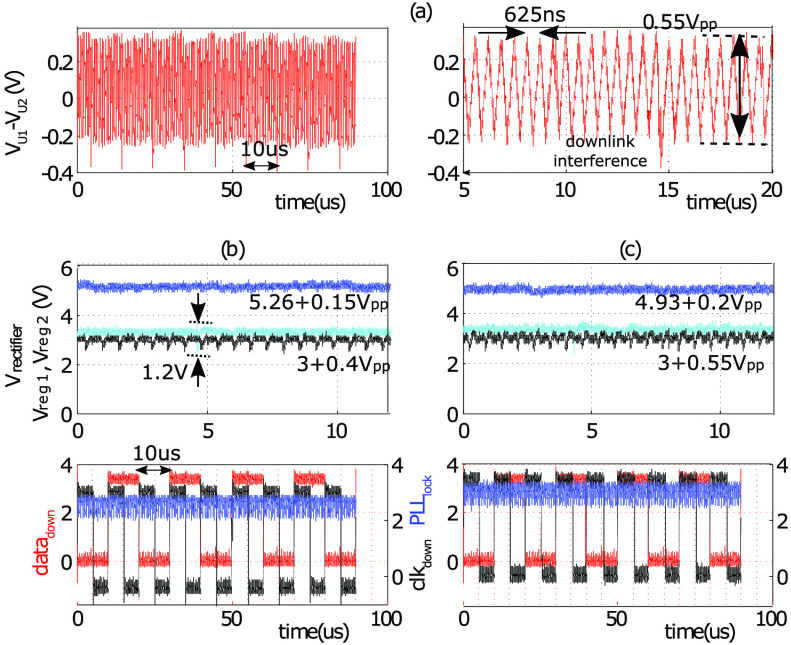
Measured waveforms when uplink and downlink are simultaneously active. (a) The received uplink, $\text{V}_{U1}$-$\text{V}_{U2}$ at 1.6 MHz, while IC_1_ drives the uplink; On the right the magnified version of the same waveform is shown. (b) The voltages at different nodes on IC_2_. (c) Same voltages on IC_1_.

The measured waveforms of downlink, power management and uplink are shown in Fig. 13; Here the frequency of $V_{down}$ was set to 50 kHz and the $PLL_{clk}$ was used to drive the uplink on IC_1_, while the uplink on IC_2_ was disabled. Results show the downlink data and clock are correctly recovered in the both devices. The bit error rate was measured during a 15 hour test (i.e. $2\times 10^{8}$ data samples) on both IC_1_ and IC_2_, and no bit error was found.

A digital isolator (ISO7320C) per optrode was used to drive the UL$_{EN}$ and UL$_{data}$ from a function generator while keeping the supply and ground domain of the function generator and the 4-wire ASIC decoupled. The isolators were powered through the voltage rectifier ($\text{V}_{down}$ amplitude was increased to accommodate for the extra load and ensure 5 V at rectifier output).

**Fig. 14.**
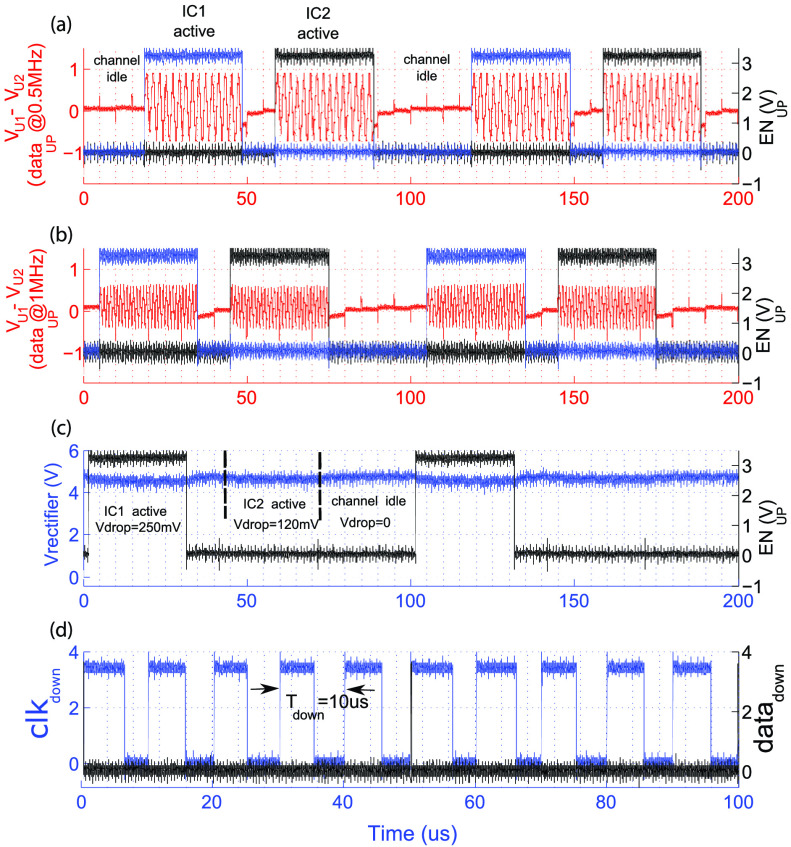
Measured waveforms at the 4-wire, power management, downlink, and uplink circuits when two 4-wire ICs are connected in parallel to the Cooper cable. (a) Received $\text{V}_{U1}$-$\text{V}_{U2}$ when Data$_{up}$ is at 500 kHz; Two non-overlapping EN$_{up}$ signals enable the uplink on two ICs; (b) same signals when Data$_{up}$ is at 1 MHz; (c) the rectifier output and uplink enable EN$_{up,1}$ of IC_1_ during uplink activity; (d) the recovered clk$_{down}$ and data$_{down}$ during uplink activity.

The differential uplink waveform received at the chest unit end of the lead was measured at different $data_{up}$ frequencies, 500 kHz and 1 MHz. Results are shown in Fig. 14–(a) and (b). Although the amplitude of the received waveform does not settle to $\text{V}_{up}$ (i.e. 3 V), it has a peak-to-peak amplitude of 0.85 V at 500 kHz and 0.55 V at 1 MHz, and can be easily detected on the chest unit.

The effect of uplink activity on the rectifier output is shown in Fig. 14–(c); The average value of the rectified voltage on IC_1_ drops by 225 mV and 112 mV during uplink activity on IC_1_ and IC_2_, respectively. The latter is due to the increased R_*s*_I drop on D1 and D2 wires. At the same time the CDR recovers the data and clock correctly from downlink (Fig. 14–(d)). The PLL however oscillates between lock and unlock conditions which could be attributed to the effect of large voltage spikes on the rectifier output which are due to the transient activities of the isolator IC during the transitions of the EN$_{UP,1}$ and EN$_{UP,2}$.

### C. Stimulation Phase

**Fig. 15.**
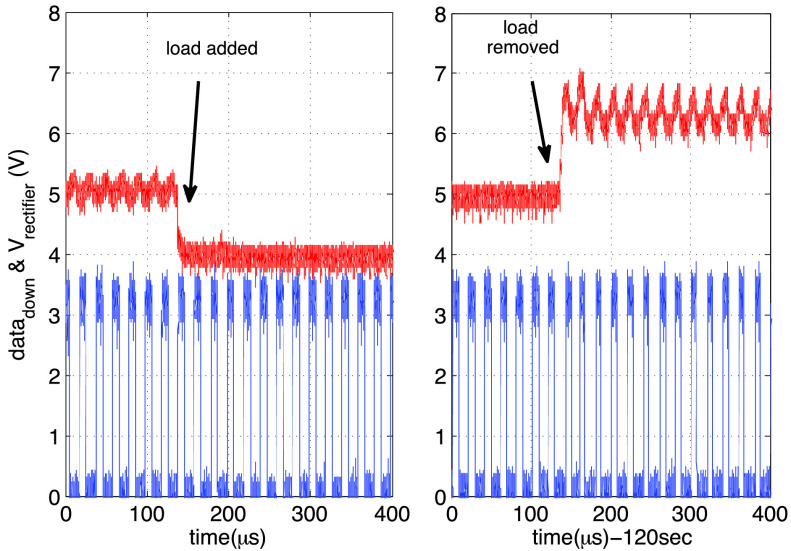
The emulated effect of LED stimulation on the recovered data and the rectifier voltage: Left: A load of 2.7 $\text{k}\Omega $ (${\tilde {1}}.8 $ mA) added to the rectifier output; right: The load is removed after 120 s; the ripples on the rectifier decay within $80~\mu \text{s}$. During the two load changes, $\text{V}_{down}$ was increased to increase the rectifier output voltage to 5 V.

The effect of LED stimulation on the 4-wire ASIC was emulated by an abrupt change in the load of the rectifier. In particular it is important that the optrode recovers downlink commands (Data$_{down}$) correctly while it is in the stimulation mode. To verify this, a resistive load of $\text{R}_{Stim} =2.7~\text{k}\Omega $ ($\simeq 1.8$ mA) was switched on and (after almost 120 s) off at the rectifier output. Fig. 15 shows the variation of the rectifier output during the load switching. When the load is inserted or removed the output voltage of the rectifier changes by almost 1 V. Part of this is due to the voltage drop on the cooper cable ($\text{R}_{s}~\times1.8$ mA = 0.57 V) and the rest (0.43 V) is due to the voltage drop on the rectifier. The latter is due to the use of the diode-connected thick-oxide PMOS devices in the rectifier which have relatively high threshold voltage and small transconductance (i.e. high resistance). In between the two transitions, the $\text{V}_{down}$ at the chest side of the lead was increased manually to maintain 5 V at rectifier output. The manual tuning is to emulate the automatic and dynamic adjusting of $\text{V}_{down}$ according to Eqn. 4 through the Chest implant. The recovered Data$_{down}$ is not affected by the load switching, indicating the correct operation of downlink during the stimulation phase.

## VIII. Conclusion

This paper has presented a fully-integrated 4-wire interface for transmitting power and full-duplex communication in intrabody implantable devices. The target application is an implantable neural prosthesis with single chest unit wired (using link presented herein) to a head unit consisting of multiple optrode devices connected in parallel. On-chip power management recovers a smooth unregulated 5 V and regulated 3.3 V and 3 V DC supplies for powering the optrode core circuits. Full-duplex communication between the chest unit and multiple optrodes is achieved providing a datarate of 100 kbps for downlink and up to 1.6 Mbps for uplink.

The measured power transmission efficiency is up to 66% and depends on the total average current of the brain implant. The uplink and downlink communication were successfully tested in a system of two-optrodes. In particular the robust CDR circuit ensures reliable data and clock recovery during the (emulated) stimulation phase. Table IV compares the proposed system with state-of-the-art systems for wired communication.

**TABLE IV:** System Characteristics and Comparison With State-of-the-Art

Parameter	[unit]	This work	[8]	[9]	[14]	[15]	[29]	[30]
Year		2017	2015	2005	2014	2013	2012	2009
#wires		4	3	4	4	2	5–8	4
#implants		1 – 16	2	2–NA	2	2	2–5	2
Distance	[cm]	77	few	~100	few	few	~100	~1
AC-coupled		Y	N	N	N	Y	N	N
Power transmission		Y	Y	Y	Y	Y	Y	Y
External components		N	Y	NA	Y	N	N	NA
CMOS technology	[nm]	350	180	–	65	350	600	350
Maximum PTE	[%]	66	NA	NA	70	NA	NA	NA
Communication		Full-duplex	Unidirectional	Semi-duplex	Full-duplex	Semi-duplex	Semi-duplex	Unidirectional
Encoding^*^ (uplink, downlink)		Mnctr, Mnctr	–,PWM	NA	PCM, Mnctr	LSK, PWM	NA, Custom	NA
Datarate (uplink, downlink)	[kbps]	1600, 100	–,500	NA	100, 600	–, 222	–,50	NA

^*^ Mnctr=Manchester coding, PCM=pulse code modulation, LSK=load shift keying,PWM=pulse width modulation

Ongoing work is focusing on developing an embedded platform for the chest unit. This will allow for dynamic voltage adjustment of $\text{V}_{down}$ depending on the load demand. This will additionally enable us to study the effect of lead interference, and long term reliability in a realistic body phantom.
